# Does inclusive leadership affect the organizational socialization of newcomers from diverse backgrounds? The mediating role of psychological capital

**DOI:** 10.3389/fpsyg.2023.1138101

**Published:** 2023-04-12

**Authors:** Xinxing Dai, Yangchun Fang

**Affiliations:** ^1^Ningbo University of Finance and Economics, Ningbo, China; ^2^Zhejiang University of Technology, Hangzhou, Zhejiang, China

**Keywords:** inclusive leadership, organizational socialization, psychological capital, newcomers, relationship

## Abstract

**Introduction:**

This paper aims to explore the influence of inclusive leadership on the organizational socialization of newcomers from diverse backgrounds, as mediated by psychological capital.

**Methods:**

Structural equation model was used by Mplus 8.3 to analyze the effect of inclusive leadership and psychological capital on organizational socialization. This study used the 1,000 bootstrap replications method and the mediating path effect test to find out the mediating effect of psychological capital between the relationship of inclusive leadership and organizational socialization.

**Results:**

The result found that (1) inclusive leadership positively affects newcomers’ organizational culture, interpersonal relationships,socialization into organizational politics and can improve employees’ job competence. (2) Psychological capital plays an intermediary role between inclusive leadership and the organizational culture, interpersonal relationships and socialization into organizational politics and job competence. Inclusive leaders improve newcomers’ psychological capital by encouraging and recognizing their diversity, tolerating their mistakes. It can increase new generation of newcomers’ resilience, self-efficacy and optimism. Individuals with high psychological capital are more likely to integrate into the organizational culture and increase their interaction with colleagues and then are more likely to integrate into the organization.

**Discussion:**

Organizations should encourage leaders to develop inclusive leadership behaviors to foster newcomer socialization. In an inclusive leadership context, newcomers have high levels of psychological capital that promote their organizational socialization. Inclusive leadership is crucial for helping newcomers from diverse backgrounds to integrate into organizations.

## Introduction

Organizational socialization (OS) is a dynamic development process by which a newcomer makes the transition from being an outsider to becoming an insider (Jokisaari and Nurmi, 2009). Organizational socialization is important for shaping the unique core competitiveness of enterprises and promoting employees to achieve good performance in the organization (Wang, 2006; Wang and Ling, 2006; Wang and Zhu, 2006). When employees are fitting in an new organization, newcomers often face considerable ambiguity, feel anxious about fitting in new organizations, and have a desperate need for information and social support to reduce role ambiguity and job uncertainty (Ashforth and Saks, 1996). However, due to the different growth environment and education background, the new generation of newcomers in China have unique characteristics, which exhibits distinct psychological characteristics and behavior patterns, like strong need for self-realization and personal interest (Wang et al., 2021). The new generation of employees generally have diversified value demands, are eager to express self-suggestions and focus on the realization of self-value, so they need tolerance and support from the organization more (Zhang et al., 2020). These special characteristics affect their work attitude and behavior performance and may lead intra-group conflicts and discrimination during the process of fitting in the organization (Kulkarni, 2015). Therefore, how to include newcomers of new generation’s during the process of OS in China is important to study.

The new generation of newcomers desire to belong and be recognized by their organization (Liu and Tang, 2019) and are anxious for more inclusion from the organization to respect their differences, encourage them to play their strengths, meet their diverse needs. Therefore, the new generation of employees may need more inclusion from organization. Organizations should identify these differences and adjust management mode, leadership styles and incentive mechanisms to accommodate employees of different generations (Kampf et al., 2017).

The OS research has traditionally emphasized the effect of organizational context on newcomers’ adjustment experiences (Ashforth et al., 2007; Saks et al., 2007). In addition to organizational context, leaders or managers play an impact and integral role in newcomers’ OS (Ashforth et al., 2007). Organizations invest heavily in orientation programs, specially the role of leaders who are essential in helping newcomers navigate the new environment. Leaders serve as interpreters of knowledge (Sluss and Thompson, 2012). They can provide the necessary resources and suggestions that enable newcomers to reciprocally interact with the new context, and ultimately become attached to it. As a new generation of employees enters the workforce, employers have acknowledged the importance of inclusive leadership (He et al., 2007; Peng et al., 2008). This leadership style focuses on integrating diverse individuals into the organization (Tang and Zhang, 2015). It allows employees to perceive the inclusive culture by recognizing employees’ unique values, respecting their differences and tolerating mistakes (Fang, 2014). Inclusive leadership is a special type of relational leadership (Carmeli et al., 2010) that can enhance employees’ insider perception (Shore et al., 2011). Despite the importance of inclusive leadership in diverse workforce, limited research has been conducted on the relationship between inclusive leadership and OS. Therefore, examining the effect of the key roles played by the inclusive leadership on newcomer OS has important theoretical and practical implications.

Psychological capital can affect leadership’s influence on employees and predict employees’ behaviors (Luthans and Youssef, 2007). Fang found that psychological capital is the mediating factor for inclusive leadership to predict employees’ behaviors (Fang and Wang, 2016). Inclusive leadership will first affect the individual’s psychological state and then lead to individual behavior changes (Nembhard and Edmondson, 2006). Leaders’ encouragement, support and trust will enhance employees’ psychological capital (Sahin et al., 2014). If a leader considers an employee’s value, needs, and motivations carefully, the employee will become more optimistic and confident in their pursuit of socialization.

This study’s primary aim was to explore inclusive leadership’s influence on a new generation’s OS. We proposed that OS can be realized by building a mediation model of the relationship between inclusive leadership and a newcomer’s OS, with psychological capital as a mediator. This study makes three primary contributions to the literature. First, OS was traditionally studied by scholars from the view of socialization tactics, ignoring the effect of leadership. This study explores the mechanism influencing inclusive leadership on newcomers’ OS in response to this trend. Second, few papers have analyzed OS from the perspective of positive psychology. This study explores the mediating role of psychological capital between inclusive leadership and newcomers’ OS, and analyzes the process of employee socialization from a psychological perspective. Third, the study enriches the research on inclusive leadership. Inclusive leadership is an important, emerging theory in the leadership research field. However, little research has studied inclusive leadership in relation to newcomers’ OS.

## Theoretical background and hypothesis development

### Organizational socialization

Newcomer socialization is the process by which individuals acquire knowledge, behaviors and attitudes to become an “insider” in an organization (Fisher, 1986). Individuals who join an organization are easily overwhelmed as they are expected to adapt to the new environment immediately. OS theory states that newcomer’s learning is the major mechanism by which newcomer fit in with the organization’s needs (Ashforth et al., 2007). Newcomer learning typically refers to the process by which new staff learn to adapt to their working environment, adjust to routines, learn necessary attitudes and skills to fulfill their roles. The learning content mainly focuses on the job or task, work group and organization in the theoretical sense (Klein and Weaver, 2000). However, during socialization, employees must learn the history, value, politics, behaviors, and working skills of the organization and understand the interpersonal relationships within it. In Chinese culture, particularly, the quality of relationships is an important factor for working successfully in the nascent stage. Therefore, Chinese scholars have analyzed the content of OS widely and developed many scales to measure it in this setting (Yu and Davis, 2015), like Zhao et al. (2007) compiled a scale composed of organizational culture, job competence, interpersonal relationships, and organizational politics based on the Chinese context.

How to socialize newly hired employees is as important as the content of socialization (Ashforth et al., 2007). Some scholars consider that situational, interactional and individual differences can explain individuals’ socialization in organizations (Saks and Ashforth, 1997; Song and Chathoth, 2010). As the importance of leadership in OS became recognized, the potential importance of relationship between leaders and employees began to receive attention (Sluss and Thompson, 2012). According to the social exchange theory, the acquisition of resources and cognitive transformation of newcomers during socialization can be considered the result of the positive relationship between leaders and newcomers (Bauer et al., 2007). Newcomers develop a high-quality exchange relationship with leaders in organization to gain support and resource. Based on the socialization resource theory (Saks and Gruman, 2012), newcomers always experience anxiety and uncertainty (Allen and Shanock, 2013) and therefore need appropriate resources and support from the organization to successfully adjust to their roles. Leaders serve as a support and resource for newcomers to experience generalized reciprocity with and attachment to the new organization (Shore et al., 2009). This manner of providing support and social resources to newcomers is consistent with the general characteristics of inclusive leaders. In the face of a new generation of employees from diverse demographic and social backgrounds, inclusive leaders can create a supportive (Carmeli et al., 2010) fairly and inclusive relationship with employees (Hollander, 2009), through which inclusive leaders help newcomers to take the initiative and participate in organizational learning and adaptation. Newcomer’s participation is likely to improve their learning and assimilation, helping them adjust to the new organization.

### Inclusive leadership and organizational socialization

Different definitions of inclusive leadership have been proposed, reflecting cultural differences between China and other countries. Nembhard and Edmondson (2006) stated that inclusive leadership is people-centered; inclusive leaders are good at listening to employees’ voices, encouraging employees to work and recognizing their contributions. Based on this concept, Carmeli et al. (2010) and his colleagues argued inclusive leadership is a special “relational leadership.” Inclusive leaders are characterized by open minds, efficient management, and availability. In traditional Chinese culture, inclusive leadership is mainly reflected in the two aspects of “Bao” and “Rong” (Tang et al., 2014). “Bao” means inclusion, consistent with western scholars, and indicates equal treatment of differences. “Rong” means tolerance, understanding of employees and forgiveness of mistakes, a unique concept of Chinese culture. Fang and Jin (2014) described the unique connotation of “rational tolerance of employee mistakes” within traditional Chinese culture. They developed an inclusive leadership scale made up of three dimensions: (1) encouragement and recognition of employees, (2) respect and fair treatment of employees, and (3) rational understanding and tolerance of employee failure. The construct of inclusive leadership has been validated in some researches (Fang and Chen, 2017). We selected the above construct of inclusive leadership for theoretical and empirical analysis in the present study.

Social exchange theory states that a high-quality relationship increases the sense of mutuality or reciprocity. The newcomers will perceive the mutual beneficial relationship and then respond positively to the resource provided on the initial stage. Inclusive leaders can build an open-ended relationship with newcomers through their encouragement and recognition. They are available to provide support and access to information that includes organizational culture, politics and related work skills for newcomers from diversity background to fulfill job requirements. The encouragement and recognition a leader provide to newcomers can build social ties and help promote interpersonal relationships with others in the organization by providing socio-emotional support (Hollander, 2009). When Newcomers perceive the inclusive and open relationship with leaders, they will increase the enthusiasm for task learning and relationship building. Inclusive leaders who respect and treat employees from diversity background fairly enhance the sense of belonging to an organization (Liu et al., 2012; Easterbrook and Vignoles, 2013). As an insider in an organization, employees can avoid relationship conflict and contradiction. The sense of belonging and strong emotional links within an organization promote internal relationship building, which accelerates organizational information sharing, like organizational culture, politics and necessary work skills. Inclusive leaders’ rational understanding and tolerance of newcomers’ failures establishes an inclusive, warm and open working atmosphere (Nishii, 2013) that fosters the broad participation of newcomers from diverse backgrounds and leads to better internal relationship building. Moreover, with inclusive leaders’ tolerant behaviors, newcomers are more willing to learn about the organization, adapt to organizational culture and politics, and try different ways to resolve problems without worrying about negative consequences. Therefore, this paper proposes the following hypotheses:

*H1*: Inclusive leadership is positively correlated with newcomers’ OS.

### Inclusive leadership and psychological capital

Psychological capital is the positive psychological resource of an optimistic attitude and motivated tendency towards work and life (Sahin et al., 2014; Ke and Sun, 2018). The most widely used structure of psychological capital was developed by Luthans and Youssef (2007), and consists of the following four factors: self-efficacy, hope, optimism and resilience. Psychological capital is an important resource and guarantee for individuals to obtain a sustainable competitive advantage in the future (Ren et al., 2013) and achieve career success and life happiness (Ke and Sun, 2018). Psychological capital can predict individuals’ job performance (Jiang and Zhao, 2007; Walumbwa et al., 2010), job satisfaction (Luthans and Youssef, 2007), and innovation (Baluku et al., 2018). Many studies have shown that psychological capital is the product of the combined effect of an individual, leadership and organization (Ke and Sun, 2018). Psychological capital can play a mediating role between leadership and individuals’ behaviors, job performance and innovation (Luthans et al., 2004; Mao, 2008).

Fang and Wang (2016) and Fang et al. (2019) found that inclusive leadership has a significant positive impact on employees’ psychological capital. According to social exchange theory, a high-quality exchange relationship between leaders and employees can produce an effective interaction that increases employees’ positive psychological state for work. Thus, if leaders treat newcomers positively and inclusively, their psychological capital is more likely to be enhanced. Positive support can be described as respect and fair treatment, encouragement and recognition, and rational understanding and tolerance of employees’ failures at work. Support from leaders can help build mutual trust and a reciprocal relationship with newcomers, leading to positive emotions for newcomers (Nembhard and Edmondson, 2006; Fang and Chen, 2017). With positive emotion and enthusiasm, newcomers are likely to be hopeful, optimistic, self-efficacious and resilient and then increase psychological capital (Fang and Chen, 2017). Moreover, inclusive leadership can create a safe working environment for employees (Carmeli et al., 2010) that allows newcomers to share novel methods and take risks to solve problems in the early stages. Thus, newcomers can be expected to have hope and optimism in adapting to their new environment, enhancing their self-efficacy and resilience to overcome difficulties while feeling secure that negative consequences will not result from such behaviors (Snyder et al., 2005).

*H2*: Inclusive leadership is positively correlated with psychological capital.

### Psychological capital as a mediator

Consistent with previous research, psychological capital is a positive psychological resource. It can be affected by leadership and serves as a social-psychological mechanism by which newcomers can integrate into an organization without experiencing negative consequences. Newcomers can use psychological capital as a resource to adjust their behaviors and attitudes to fit in an organization (Snyder et al., 2005). Those with high psychological capital are more likely to take the initiative and become broadly involved in organizational activity, which is ultimately important for the experience of OS. Psychological capital enables newcomers to engage in the socialization process. Adaptation, integration and learning are more likely to occur when newcomers have a high degree of psychological capital from the outset of their employment.

Inclusive leaders convey positive emotions and provide support to improve newcomers’ psychological capital through encouragement and recognition that stimulates newcomers’ internal motivation (Zeng and Zhao, 2007). Individuals with high internal motivation are more likely to recognize opportunities, take the initiative to integrate into the organizational culture and increase their interaction and communication with colleagues. They can then share work skills, improve interpersonal relationships, and obtain more information about their organization’s politics. Inclusive leaders increase employees’ sense of belonging to the organization by treating employees with respect and fairness so that they will not feel excluded (Duan and Tang, 2016). The stronger the sense of belonging, the higher the expectation of organizational adaptation. Moreover, a stronger sense of belonging will improve the initiative to participate in organizational activities and the communication among employees. This will then, in turn, increase the mutual trust and information sharing (like work skills, organizational politics and other organizational knowledge) among employees. Inclusive leadership supports employees by offering rational tolerance for mistakes. It stimulates employees’ psychological capital, helping them believe they are capable of mastering skills to complete organizational tasks without worries. They are then more likely to integrate into the organization, improve interpersonal relationships, adapt to the organizational culture and share in organizational politics. Therefore, the following hypotheses were proposed:

*H3*: Psychological capital has a mediating role in the relationship between inclusive leadership and newcomers’ OS.

## Methods

### Participants and procedure

The whole investigation process of this study is divided into two stages: (1) Firstly, the employment guidance centers of universities in South China to determine the new graduates who have been in the enterprises for 6 months, the formal organizational socialization scale and psychological capital scale are used to conduct the first survey on them; (2) The second survey was conducted in the next month after the first survey, The selected investigator was asked to fulfill the inclusive leadership scale. According to the results of relevant theoretical and empirical research, the stage division of individual organizational socialization, can be divided into three stages: Prearrival Stage Stage, Adaptive Stage and Metamorphosis Stage (Feldman, 1976; Wanous, 1992; Kammeyer-Mueller et al., 2011). Prearrival Stage period refers to the period when an individual learns about the organization or the position he or she is engaged in (values, attitudes and expectations). The adaptation period refers to the period of acquisition of cognition and experience of the whole organization. The metamorphosis period refers to the period of repositioning, adjustment and change, and forming deeper cognition and experience of the organization. The adaptation period and the metamorphosis period have attracted more attention from researchers. Previous literature has found that relevant foreign studies usually determine the survey points of the adaptation period as 4–6 months after entry (Cable and Judge, 1997; Cable and Parsons, 2001; Fang et al., 2011). Therefore, this study determine the time period of the two surveys around the sixth months and the 7 month after they enter the enterprises.

The respondents in the research were anonymous and agreed to participate in this study and completed the questionnaire voluntarily. This survey was conducted online in Chinese. None of the questions involved confidential information and did not relate to any sensitive issues. A total of 180 valid questionnaires were collected, and 178 were successfully matched, with an effective response rate (74%). The ratio of male to female respondents was 36 and 64%, respectively. Employees under the age of 25 accounted for 65%. Of these, more than half (82%) had a bachelor’s degree (Table 1).

**Table 1:** Demographic data.

Characteristic	Categories	Frequency	Percentage (%)
Gender	Male	64	35.96
	Female	114	64.04
Age	20–25 years	116	65.17
	25-30 years	54	30.34
	30 years and over	8	4.49
Education	College	1	0.56
	Bachelor	146	82.02
	Master and over	31	17.41
Position	Administration department	4	2.25
	Finance department	147	82.58
	Sales department	13	7.30
	Technical department	2	1.12
	others	12	6.74

### Measures

All surveys were conducted in Chinese, and variables were assessed on a 5-point Likert scale where 1 is “strongly disagree,” and 5 is “strongly agree.”

*Organizational socialization*: There is controversy about the content of organizational socialization. In Chinese culture, particularly, the quality of relationships is an important factor for working successfully in the nascent stage. Therefore, Chinese scholars have analyzed the content of OS widely and developed many scales to measure it in this setting (Yu and Davis, 2015). Zhao et al. (2007) compiled a scale composed of organizational culture, job competence, interpersonal relationships, and organizational politics based on the Chinese context. The scale has been widely validated by scholars in Chinese organizations (Hu, 2014; Zhang, 2019). Thus, the present study used this scale to analyze the organizational socialization of newcomers.

*Inclusive leadership*: We used Fang (2014) definition of inclusive leadership consisting of three parts (1) leaders’ encouragement and recognition of employees, (2) leaders’ respect and fair treatment of employees, and (3) leaders’ rational understanding and tolerance of employee failure.

*Psychological capital*: This paper adopted the psychological capital scale developed by Luthans and Youssef (2007), and Scott and Bruce (1994), which is recognized by the majority of scholars in the field.

*Control variable*: This paper used gender, age, department, educational background, and length of employment as control variables.

## Analysis and result

Firstly, in this paper SPSS 32.0 and Mplus 8.3 were used for statistical analysis of the data. Then SPSS 32.0 was used for statistical analysis of the mean value, standard deviation, correlation and reliability of measurement of each variable. For Numerical variable, Mean is used to describe and for the Categorical variable, Frequency and Percent is used to describe. In this study, the Harman single factor test was used as the common method variance test, and Cronbach’s Alpha was used as the reliability index. In addition, this study explored the reliability and validity of the scale through exploratory factor analysis (EFA) and confirmatory factor analysis (CFA). Composite reliability (CR) and average variance extracted (AVE) were used to detect the combination reliability and convergence validity, respectively. Correlation analysis was conducted to investigate the relationship between variables. Finally, in this paper, structural equation model was used by Mplus 8.3 to analyze the effect of inclusive leadership and psychological capital on organizational socialization. This study used the 1,000 bootstrap replications method and the mediating path effect test to find out the mediating effect of psychological capital between the relationship of inclusive leadership and organizational socialization.

### Reliability and validity tests

We used EFA to test the scales’ factor loading and reliability. CR and Cronbach’s alpha to evaluate internal consistency. The results show the scales’ every dimension’s factor loading are all over 0.6, which indicates that the reliability of the scales is acceptable. Moreover, Cronbach’s alpha and CR scores are greater than 0.70, meaning that they were both adequate. We used average variance extraction (AVE) to test the convergent validity and found AVE scores were all greater than 0.50, which means the reliability and convergent validity conform to the required standard (in Table 2). Mplus 8.3 was used to calculate the correlation coefficient and every extracted root of AVE. Table 3 shows that every extracted root was greater than the figures in rows and columns, proving the scales have good convergence and discriminant validity. Additionally, in order to avoid the problem of common method variance. The questionnaire was divided into two stages, which can be confirmed with Harman’s single-factor test. We put all variables into an EFA procedure with the principal axis factoring method (Podsakoff and Farh, 1989). For the unrotated factor part, the first one explaining 28% (<40%) of the variance. Thus, no single factor was dominant.

**Table 2:** Reliability and convergent validity analysis.

Variable	Cronbach’s α	CR	AVE
Inclusive leadership	0.91	0.93	0.82
Psychological capital	0.95	0.92	0.73
Organizational socialization	0.94	0.91	0.70

**Table 3:** Descriptive statistics of scales.

Variable	Mean	SD	Maximum value	Least value	Skewness	Kurtosis
Inclusive leadership	3.984	0.366	5	1.800	−0.957	8.313
Organizational socialization	4.035	0.371	5	2.940	−0.227	1.674
Psychological capital	3.823	0.331	5	2.880	0.438	1.816

We performed a series of CFA to assess the discriminant validity and structural validity of our measures. We compared a three-factor model with two alternative models to test the structural validity of each measurement tool. Structural validity can reflect the degree of consistency between the content measured by the scale and its own theoretical structure content. If the fitting index of structural validity reaches the minimum standard, it can be considered to have good validity. The results are shown in the table below. Assuming that the values of CFI, TLI and RMSEA of the model and each scale meet reasonable standards, it indicates that the structural validity of each scale is good, the model fit is up to standard, and the model is acceptable. Moreover, CFA was also used to conduct model verification of inclusive leadership, psychological capital and organizational socialization. The fitness indices of the model are shown in Tables 4, 5.

**Table 4:** Confirmatory factor analysis results.

model	*χ^2^*	df	*χ*^2^/df	RMSEA	TLI	CFI	SRMR
Inclusive leadership	85.41	41	2.08	0.08	0.97	0.98	0.03
Psychological capital	410.98	246	1.67	0.06	0.95	0.96	0.05
Organizational socialization	185.89	98	1.90	0.07	0.96	0.97	0.05

**Table 5:** Fit Parameters of the Hypothetical Model.

Model	*χ*^2^/df	RMSEA	NFI	TLI	IFI	CFI
Three factors model F1 + F2 + F3	1.28	0.031	0.909	0.976	0.979	0.979
Two factors model F1 + F2 + (F3)	1.33	0.033	0.905	0.972	0.974	0.974
Single factor model(F1 + F2 + F3)	1.47	0.040	0.894	0.960	0.963	0.963

### Correlation test

The Pearson correlation results in Table 3 show that inclusive leadership (comprising three dimensions), psychological capital and organizational socialization (comprising four dimensions) are significantly correlated. The three dimensions of inclusive leadership are positively correlated with psychological capital’s four dimensions. The four dimensions of psychological capital are also significantly positively correlated with the four dimensions of employee organizational socialization (see Table 6).

**Table 6:** Descriptive statistics and correlation matrix.

Variable	Mean	SD	ER	RES	TOR	HOPE	RE	SE	OP	OC	JC	IR	OP
ER	4.03	0.43	*0.87*										
RES	4.07	0.50	0.55^**^	*0.96*									
TOR	3.85	0.50	0.22^**^	0.41^**^	*0.89*								
HOPE	3.77	0.48	0.35^**^	0.35^**^	0.30^**^	*0.88*							
RE	3.72	0.50	0.27^**^	0.31^**^	0.34^**^	0.73^**^	*0.82*						
SE	3.91	0.32	0.31^**^	0.28^**^	0.28^**^	0.46^**^	0.40^**^	*0.81*					
OP	3.89	0.38	0.34^**^	0.35^**^	0.39^**^	0.43^**^	0.45^**^	0.28^**^	*0.84*				
OC	4.05	0.38	0.42^**^	0.55^**^	0.39^**^	0.43^**^	0.31^**^	0.43^**^	0.28^**^	*0.86*			
JC	4.12	0.45	0.31^**^	0.47^**^	0.33^**^	0.33^**^	0.27^**^	0.44^**^	0.29^**^	0.64^**^	*0.86*		
IR	4.04	0.44	0.35^**^	0.35^**^	0.26^**^	0.33^**^	0.34^**^	0.46^**^	0.33^**^	0.53^**^	0.60^**^	*0.86*	
OP	3.94	0.53	0.36^**^	0.41^**^	0.35^**^	0.33^**^	0.23^**^	0.42^**^	0.34^**^	0.50^**^	0.55^**^	0.61^**^	*0.87*

**p < 0.05. The italic number on the main diagonal is the square root of AVE. ER: leaders’ encouragement and recognition of employees, RES: leaders’ respect and fair treatment of employees, TOR: leaders’ rational understanding and tolerance of employees’ failures. HOPE, RE, SE and OP represent: hope, resilience, self-efficacy, optimism. OC, organizational culture; JC, job competence; IR, interpersonal relationship; OP, organizational politics. Sample size: 178.

### Hypothesis testing

**Figure 1:**
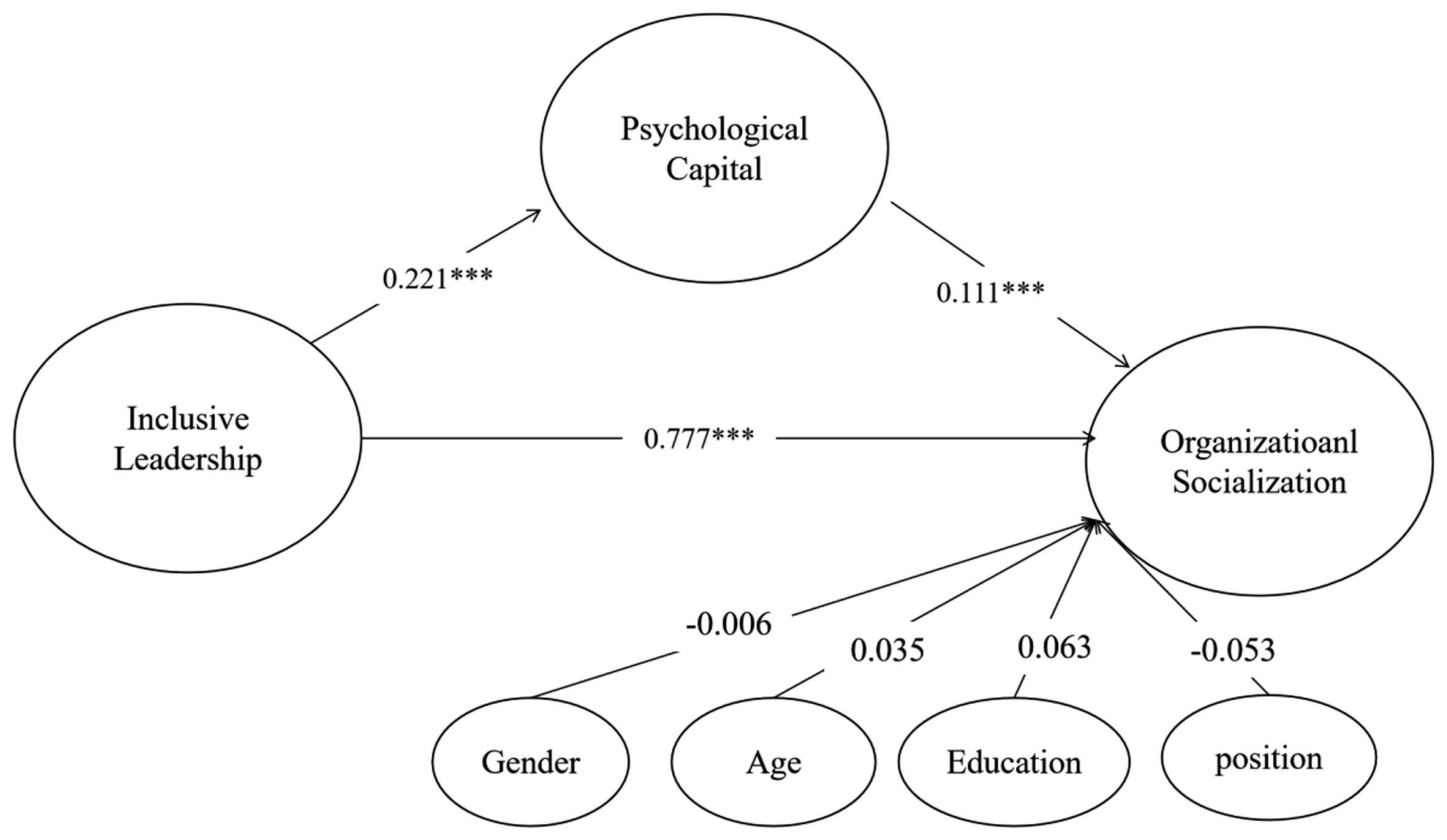
Results of the research model.

In this study, Mplus8.3 software was used to test the relationship between inclusive leadership, psychological capital and the organizational socialization of newcomers at the individual level. It can be found that *H1*, *H2* all pass the test, that is, inclusive leadership has positive effect on organizational socialization of newcomers (*H1*:β = 0.777, *p* < 0.05), inclusive leadership affect psychological capital significant (*H2*:β = 0.221, *p* < 0.05; Table 7; Figure 1).

**Table 7:** Hypothesis Test Result.

Path	Estimation		
	Effect	SE	*p*-value
Inclusive leadership → Organizational socialization (H1)	0.777	0.097	0.000
Inclusive leadership → Psychological capital (H2)	0.221	0.101	0.000
Psychological capital → Organizational socialization	0.111	0.029	0.000
Inclusive leadership*Psychological capital → Organizational Socialization (H3)	0.191	0.120	0.000

This study used the bootstrapping method to analyze the mediating role of psychological capital. Table 8 shows that the study generated 12 paths to predict the mediating effect. This indicates that psychological capital plays a strong mediating role in the three dimensions of inclusive leadership and four dimensions of organizational socialization with a 95% confidence interval. However, psychological capital fails to mediate between leaders’ encouragement and recognition of employees, and job competence at a 95% confidence interval.

**Table 8:** Mediating effect analysis.

95% Confidence interval					
The path	Estimate	S.E.	Est./S.E.	The lower	The higher
ER-PC-OC	0.12	0.04	3.70	0.06	0.22
ER-PC-JC	0.02	0.06	0.24	−0.09	0.15
ER-PC-IR	0.15	0.05	3.86	0.08	0.25
ER-PC-OP	0.16	0.05	3.38	0.08	0.27
RES-PC-OC	0.09	0.03	3.38	0.04	0.16
RES-PC-JC	0.10	0.04	3.12	0.03	0.18
RES-PC-IR	0.14	0.04	3.94	0.07	0.23
RES-PC-OP	0.13	0.04	3.21	0.06	0.22
TOR-PC-OC	0.12	0.05	3.82	0.05	0.22
TOR-PC-JC	0.13	0.05	3.59	0.05	0.25
TOR-PC-IR	0.15	0.06	4.16	0.07	0.29
TOR-PC-OP	0.14	0.06	3.44	0.06	0.28

ER: leaders’ encouragement and recognition of employees; RES: leaders’ respect and fair treatment of employees; TOR: leaders’ rational understanding and tolerance of employees’ failures. PC, psychological capital; OC, organizational culture; JC, job competence; IR, interpersonal relationship; OP, organizational politics; PC, psychology capital. Sample size: 178.

## Discussion and conclusion

In order to cope with the ever-changing economic environment, organizations need employees to quickly integrate into the enterprise, complete the transformation from outsiders to insiders in a short time. However, when the new generation of employees with unique characteristic as the main force entering all walks of life, they are easy to cause communication barriers among employees, interpersonal conflicts and other problems, affecting the effective organizational socialization process for them.

According to social cognition theory, the process of organizational socialization is influenced by the interaction among employee behavior, individual characteristics (individual differences) and situational factors (Bandura, 1986; Ostroff and Kozlowski, 1992; Yao and Le, 2008). Individual attitudes, psychology and behaviors are the result of cognition of the surrounding environment (Zhao, 2017). As an situational factor, inclusive leadership plays an important role in the adjustment of behaviors and attitudes in the process of organizational socialization of employees (Wang and Zhu, 2012). During the process of organizational socialization, the new generation of newcomers definitely need encouragement, recognition, tolerance and support from the leaders. New generation of newcomers hope leaders can accommodate the characteristics of all kinds of employees, and meet their diverse needs. Employees can form the overall impression of the organization, department and work, reduce uncertainty, enhance self-confidence, and make proactive behaviors more effective (Morrison, 2002; Wang and Zhu, 2006), so as to accelerate the process of organizational socialization.

Inclusive leadership integrates the concept of inclusiveness into all aspects of management, expresses inclusiveness, respect and encouragement of diversity for the new generation of newcomers (Fang et al., 2019), thus improving the psychological capital of employees (Fang and Wang, 2016), making them willing to actively learn organizational information and participate in group work without worrying about the punishment brought by wrong behavior. Specifically, Inclusive leadership can positively improve newcomers’ socialization into the organizational culture, interpersonal relationships, organizational politics and job competence through the behavior of encourage and recognize newcomers, treat them with respect and fairness, and understand and tolerate failure made while learning. Inclusive leaders’ encouragement and recognition, respect and fair treatment, and understanding and tolerance of failure significantly affect newcomers’ psychological capital.

Psychological capital plays a mediating role between the three dimensions of inclusive leadership and organizational culture, interpersonal relationships and organizational politics socialization. Psychological capital plays a mediating role between inclusive leaders’ respect and fair treatment of employees and job competence, and has a mediating role between leaders’ rational understanding and tolerance of employees’ failure and job competence. Inclusive leaders improve newcomers’ psychological capital by recognizing their unique characteristic and encouraging them to express themself, and increase their internal motivation (Zeng and Zhao, 2007). Individuals with high internal motivation are more likely to take the initiative to integrate into the organizational culture and increase their interaction with colleagues to obtain more information about organization’s politics. Inclusive leaders treat employees with respect and fairness so that they will have strong belong (Duan and Tang, 2016) and adapt to the new environment effectively. Inclusive leadership tolerates employees’ mistakes to stimulates employees’ psychological capital, helping them believe they are enable to complete organizational tasks. They are then more likely to integrate into the organization.

### Theoretical contributions

First, this study contributes to the literature on the antecedents of newcomers’ OS. Leadership is an important influencing factor of OS. However, few studies have analyzed leadership factors. In the face of a diverse workforce, this study introduced the theme of inclusive leadership and analyzed its influence on newcomers’ OS from relationship perspective. We thus extended the literature on leadership effects to newcomers’ OS. Second, the study considered the influence mechanism of OS from the perspective of psychological capital. Psychology is an effective way to estimate employees’ behavior in an organization. This study explored the mediating role of psychological capital and analyzed its function in the process of OS. Finally, as an emerging leadership style, there is limited research on inclusive leadership. This study considered the inclusive leadership theory in a Chinese context to enrich research on inclusive leadership.

### Practical implications

Inclusive leadership can enhance newcomers’ psychological capital to promote employees’ interpersonal relationships, integration of organizational culture and adoption of organizational politics. Organizations should encourage leaders to recognize the diversity value of newcomers and encourage them to take the initiative to integrate actively into organizations. Leaders should respect all employees, treat them fairly irrespective of their background, and provide them with equal access to organizational resources. Meanwhile, leaders need to bear the cost of their employees’ mistakes, especially in the early stages of employment, and encourage new hires to improve their work skills by themselves.

### Limitations and future directions

This research has some limitations. First, although data were collected in two-stages from the same source, it would be valuable to collect data more dynamically for analysis in the future, i.e., in an ongoing way rather than only at two time points. Second, the site for recruitment was limited to one university and the sample size was small. In the future, the sample size could be expanded to be more representative of industry characteristics, regional distribution and other aspects.

## Data availability statement

The original contributions presented in the study are included in the article/supplementary material, further inquiries can be directed to the corresponding author.

## Ethics statement

The studies involving human participants were reviewed and approved by the Ningbo University of Finance and Economics. The patients/participants provided their written informed consent to participate in this study.

## Author contributions

XD is the main author, who wrote the whole article and conduct the research process. While, YF as the supervisor who help XD to direct the research process. All authors contributed to the article and approved the submitted version.

## Funding

Ningbo Key Research Base for Philosophy and Social Studies “Regional Open Cooperation and Free Trade Zone Research Base.” This research was supported by the National Social Science Fund Project of China (Grant No. 20BGL143), the University Social Science Fund Project of Zhejiang University of Technology (Grant Nos. SKY-ZX-20200121 and SKY-ZX-20200308).

## Conflict of interest

The authors declare that the research was conducted in the absence of any commercial or financial relationships that could be construed as a potential conflict of interest.

## Publisher’s note

All claims expressed in this article are solely those of the authors and do not necessarily represent those of their affiliated organizations, or those of the publisher, the editors and the reviewers. Any product that may be evaluated in this article, or claim that may be made by its manufacturer, is not guaranteed or endorsed by the publisher.
